# Anti-mullerian hormone as a predictor of ovarian reserve and ovarian response in IVF women from Gaza strip

**Published:** 2013-04

**Authors:** Maged Mohammed Yassin, Fadel Akram Sharif, Mohammed Marwan Laqqan

**Affiliations:** 1 *Department of Physiology, Faculty of Medicine, The Islamic University of Gaza, Gaza Strip, Palestine.*; 2 *Department of Molecular Biology, Faculty of Sciences, The Islamic University of Gaza, Gaza Strip, Palestine.*; 3 *Lecture at Faculty of Medicine, The Islamic University of Gaza, Gaza Strip, Palestine.*

**Keywords:** *Anti-mullerian hormone*, *Ovarian reserve*, *In vitro fertilization*, *Gaza Strip*

## Abstract

**Back** **ground:** Careful evaluation of patients and proper treatment with right techniques are essential for successful outcome of assisted reproduction. To obtain satisfactory results, it is necessary to assess ovarian reserve before planning treatment.

**Objective:** To evaluate anti-mullerian hormone as a predictor of fertility potential in terms of ovarian reserve and ovarian response reflected by antral follicles and mature oocyte counts in response to menotrophin stimulation in in vitro fertilization (IVF) women from Gaza Strip.

**Materials and Methods:** This prospective cohort study consisted of 81 women (mean age 28.7 years) attending IVF at Al-Basma Fertility Center in Gaza City. Blood withdrawal for antimullerian hormone measurement was performed in all the patients and the number of oocytes and embryos were recorded.

**Results:** The total number of retrieved oocytes was inversely associated with age (12.5±4.5, 11.0±5.4 and 6.9±4.7 at age ≤25, 26-35 and >35 years, respectively (F=4.793 and p=0.011). The ovarian response to Menotrophin (FSH 75IU, LH 75 IU) stimulation was better for younger age. There was a significant positive association between ovarian response in terms of total number of oocytes and antimullerian hormone levels. The maximum level of antimullerian hormone was observed in females who achieved positive pregnancies (4.5±2.5 ng/mL) followed by negative pregnancies (2.9±1.8 ng/mL) with significant differences (F=6.862 and p=0.002). Correlation coefficient revealed that the number of mature oocytes showed strong positive correlation with the antimullerian hormone levels (r=0.469, p=0.001).

**Conclusion:** Anti-mullerian hormone can be used in IVF programs as a good predictor of ovarian reserve and ovarian response.

## Introduction

Careful evaluation of patients and proper treatment with right techniques are essential for successful outcome of assisted reproduction. To obtain satisfactory results, it is necessary to assess ovarian reserve before planning treatment. Contemporary markers for ovarian reserve include age and anti-mullerian hormone (AMH) levels. "AMH also known as mullerian-inhibiting substance or mullerian-inhibiting factor is a glycoprotein dimer composed of two 72 KDa monomers linked by disulfide bridges (1). AMH belongs to the transforming growth factor-β superfamily and binds to AMH receptor 2. The superfamily includes TGF-B and the various inhibin and activin glycoproteins. All members of this superfamily are dimeric glycoproteins (2, 3). AMH is produced by ovarian granulosa cells in female. After puberty, when menstrual cycling begins, circulating AMH slowly decreases throughout life and becomes undetectable at menopauses (4, 5). 

"AMH continues to be expressed in the growing follicles in the ovary until they have reached the size and differentiation state at which they are to be selected for dominance. In humans, this occurs at the antral stage when the follicle size is 4-6 mm. "The receptors of AMH, like other glycoprotein hormones, are serpentine receptors coupled to G_s_ and adenylyl cyclase signal transduction system (5-7)". AMH seems to act only in the reproductive organs (8). 

The most striking effect of AMH is its capacity to induce regression of the mullerian ducts, the anlage of the female internal reproductive organs. In the absence of AMH, mullerian ducts of both sexes develop into uterus, fallopian tubes and the upper part of the vagina (2, 8, 9). "AMH has two sites of action; it inhibits initial follicle recruitment and, inhibits FSH-dependent growth and selection of preantral and small antral follicles"(10). AMH can be used for evaluating fertility potential and ovarian response in in vitro fertilization (IVF) serum AMH levels correlate with the number of early antral follicles. 

This makes it useful for prediciting ovarian response in an IVF cycle. "Women with low AMH levels are more likely to be poor ovarian responders to gonadotrophin stimulation, and ovarian ageing-diminished ovarian reserve, is signaled by reduced baseline serum AMH concentrations. Women with poor ovarian reserve who have entered the menopause have low levels of AMH (11, 12). This study was designed in order to assess AMH in early follicular phase as a predictor of ovarian reserve among females undergoing IVF in the Gaza Strip.

## Materials and methods

This prospective cohort study consisted of 81 women undergoing IVF/ICSI program, aged between 20-40 years without history of other diseases. The subject was recruited from Al-Basma Fertility Center in Gaza City in the period October 2009 to January 2010 by used the non probability convenience sampling method. Each patient gave informed consent to participate in the study. The criteria for inclusion were as follows: (i) age 20-40 years, (ii) regular menstrual cycle, (iii) not on hormone therapy for three months and (iv) have not been subject to surgical operation in the reproductive system. This study was approved by the Ethical Committee of the institution. All participants were guaranteed confidentiality, and only the principal investigator has full access to the data.

**Sonography, blood sampling and IVF protocol**

The vaginal sonography was performed in the second day of the menstrual cycle. Blood samples were collected on the third day of the menstrual cycle. Clear serum samples were then separated by centrifugation at 3500 rpm for 10 minutes and stored at -20^o^C until use. All serum samples were submitted to AMH determination using Diagnostic Systems Laboratories Inc ELISA kit for AMH. Estradiol (E_2_) levels were determined using TECO Diagnostics ELISA kit for E_2_. 

There were two protocols used during ovarian hyperstimulation, the first one is long protocol, which relies on pre-stimulation pituitary down regulation using GnRH agonists in daily intermittent or depot formulations, the second one is short protocols using GnRH antagonist during the late follicular phase of the stimulation cycle have been utilized. Adding recombinant LH to recombinant FSH protocols, when starting antagonists, as a strategy to increase oocyte yield and improve pregnancy rates. Human chorionic gonadotropin (hCG) was injected at a dose of 5000 or 10,000 IU. Oocyte retrieval for IVF was then typically scheduled for 30-34 hr thereafter. Then the fertilized oocyte was placed in G1 media for 3-4 days then in G2 media before rewind. 

**Statistical analysis**

Data were computer analyzed using SPSS/ PC (Statistical Package for the Social Science Inc. Chicago, Illinois USA, version 13.0). Simple distribution of the study variables and cross tabulation were applied. One-way ANOVA test was used for evaluating the relation between hormone level and qualitative variables. Correlation coefficient (r) between the number of mature oocytes and the different parameters investigated was used. The results in all the above mentioned procedures were accepted as statistically significant when the p-value was less than 5% (p<0.05).

## Results

The study populations consisted of 81 females who were seeking IVF at Al-Basma Fertility Center, Gaza Strip. Medical records showed that the infertility etiology was mostly referred to male infertility 58 patients, (71.6%), The IVF outcome was positive in 33 (40.2%) and negative in 39 (48.1%) of the cases. However, IVF showed no cleavage (no embryo) in 9 (11.1%) of the cases. The mean age of the enrolled cases was 28.7±5.4 years. According to the number of oocytes retrieved upon stimulation by menotrophin (FSH 75 IU, LH 75 IU), the patients were divided into poor, normal, good and high responders as indicated in the Table I. 

The increase in the mean levels of AMH paralleled the increase in the total number of oocytes, showing 1.0±0.5 ng/mL, 2.3±1.8 ng/mL, 3.7±1.8 ng/mL and 5.90±2.9 ng/mL in poor, normal, good and high responders, respectively. This successive change was found to be significant (F=9.174 and p=0.000). Similar trend was observed for the mean number of mature oocytes with (F=53.738 and p=0.000) and a significant inverse relationship was found between the total number of oocytes and age (F=4.934 and p=0.003). This indicates that younger women yield more oocytes. The relationship between IVF outcome, indicated by β-hCG hormone test, and the parameters investigated is provided in table II. 

IVF outcome showed that positive pregnancy occurred in women aged 27.4±4.8 years whereas negative pregnancy and no cleavage occurred at ages 28.1±5.7 and 32.3±5.5 years, respectively. When related to age, IVF outcome showed that the chance of IVF success increased with decreased age (F=3.077 and p=0.05). The maximum mean level of AMH was observed in positive pregnancy (4.5±2.5) and the mean number of mature oocytes decreased among different classes of IVF outcome showing the mean values: 10.5±3.9 in positive pregnancy, followed by 7.3±3.5 in negative pregnancy and by 4.8±4.1 in "no cleavage". The difference between these three classes of IVF outcome was found to be significant (F=11.262, p=0.000). When the relationship between the number of mature oocytes collected and the investigated parameters was analyzed, only AMH and number of embryos showed positive correlation with the number of mature oocytes. Strong but negative correlation was observed between age and E_2_ with mature oocytes (Table III).

**Table I:** Ovarian response of women attending IVF program in relation to investigated parameters

**Variable**	**<4 oocytes** **(Poor responders)** **(n=2)**	**4-8 oocytes** **(Normal responders)** **(n=26)**	**9-16 oocytes** **(Good responders)** **(n=43)**	**> 16 oocytes** **(High responders)** **(n=10)**	**F**	**p-value**
Age (year)	36.5 ± 5.0	30.6 ± 5.9	27.0 ± 4.5	26.3 ± 5.2	4.934	0.003
AMH (ng/mL)	1.0 ± 0.5	2.3 ± 1.8	3.7 ± 1.8	5.90 ± 2.9	9.174	0.000
E_2_ (pg/ml)	63.7 ± 4.9	38.1 ± 18.3	32.9 ± 12.0	34.0 ± 13.4	3.313	0.024
Number of mature oocytes	2.0 ± 0.0	4.2 ± 2.4	9.7 ± 2.1	14.1 ± 3.7	53.738	0.000
Number of immature oocytes	0.5 ± 0.7	2.0 ± 1.3	2.7 ± 2.2	6.3 ± 3.8	10.453	0.000
Number of embryos	0.0 ± 0.0	2.3 ± 1.7	4.1 ± 1.4	4.5 ± 0.7	13.694	0.000

All values are expressed as mean±SD.                     P<0.05 was considered for statistical significance.

F: ANOVA test.                                                       AMH: Anti-mullerian hormone.

E_2_: Estradiol.

**Table II:** IVF outcome in relation to the investigated parameters

**Variables**	**Positive** **(n= 33)**	**Negative** **(n= 39)**	**No cleavage** **(n= 9)**	**F**	**p-value**
Age (year)	27.4 ± 4.8	28.1 ± 5.7	32.3 ± 5.5	3.077	0.050
AMH ( ng/mL)	4.5 ± 2.5	2.9 ± 1.8	2.1 ± 1.5	6.862	0.002
E_2_ ( pg/ml)	32.8 ± 10.1	35.6 ± 17.4	44.5 ± 17.2	2.771	0.121
Number of mature oocytes	10.5 ± 3.9	7.3 ± 3.5	4.8 ± 4.1	11.262	0.000
Number of immature oocytes	3.1 ± 2.4	2.8 ± 2.9	1.6 ± 1.9	0.817	0.446
Total number of oocytes	13.7 ± 5.1	9.8 ± 4.3	6.7 ± 4.1	10.820	0.000
Number of embryo	0.8 ± 4.5	3.4 ± 1.5	0.0 ± 0.0	50.561	0.000

All values are expressed as mean±SD                    P<0.05 was considered for statistical significance.

F: ANOVA test.                                                     AMH: Anti-mullerian hormone.

E_2_: Estradiol.                                                          Positive: pregnancy occurred. Negative: no pregnancy.

**Table III:** Correlation between the number of mature oocytes collected and the different investigated parameters

**Variable**	**r**	**p-value**
Age	-0.355	0.001
AMH ( ng/mL)	0.469	0.001
E_2_ ( pg/ml)	-0.248	0.025
Number of embryos	0.512	0.001

P<0.05 was considered for statistical significance.                    r: Correlation coefficient.

AMH: Anti-mullerian hormone.                                                 E_2_: Estradiol.

## Discussion

"Data presented in this study dealt with 81 women enrolled in IVF programs. The mean age of the cases in the present study (28.7 years) was close to that reported in Egyptian (29 years) and Iranian (29.1 years) studies (13-16), but lower than that reported from the Netherlands (33.8 years)" (5, 16). The younger age of women seeking IVF in developing countries, including Gaza Strip, could be explained in the context of social habits where people marry at young age and the desire to have children immediately after marriage. Women undergoing IVF showed that ovarian response is better with decreasing age, showing poor response (<4 oocytes) at the oldest age (36.5 years) and high response (>16 oocytes) at the youngest age (26.3 years). Consequently younger women have a better chance of a successful IVF. This inverse relationship between women response and age was also documented in other studies (15-19). 

The present study illustrated that the mean levels of AMH showed a progressive increase parallel to the total number of oocytes in poor, normal, good and high responders. Several investigators have reported similar successive increase in the total number of oocytes as a result of increasing AMH levels in responder women attending IVF (3, 15, 18-21). In contrast to AMH, the total number of oocytes in different response classes increased with decreased levels of E_2_. This inverse relation was also reported by, but with no significant change, indicating that E_2_ is a poor marker of ovarian response (16, 17). The mean number of mature and immature oocytes and number of embryos were significant in different classes of responders to ovarian stimulation from poor to high responders. This finding is in agreement with that found by who reported that the mean number of oocytes was significantly lower in poor respondent women than in normal, good and high respondent women attending IVF programs (3, 15, 18, 22). This leads to the conclusion that the ovarian response can be regarded as a reflection of the ovarian reserve.

IVF results presented in this study showed that the chance of IVF success increased with decreased age; 33 (40.7%) of cases ending up with pregnancy were 27.4 years old. This implies that younger women had a better chance to have a successful pregnancy. The results of IVF classes also showed that the maximum level of AMH was observed in the cases which ended up with positive pregnancy, followed by negative pregnancy and "no cleavage", with significant difference in AMH levels among these three classes (22-24). The observed high mean levels of AMH can be linked to the increased mean number of collected oocytes and consequently to increased chance of obtaining higher mean numbers of embryos. The ultimate outcome is to improve the chance of pregnancy (25). 

This implies that AMH levels may be an indicator for ovarian responsiveness, it is worth mentioning here that although AMH level seems to be a good marker for ovarian response to stimulation drug IVF it can't however, predict the outcome of the IVF process since 40.7% of the cases with elevated AMH did not yield a viable pregnancy recorded a higher mean level in AMH in pregnant women undergoing IVF as compared to those who failed and came to the similar conclusion that AMH predicts ovarian responsiveness (23, 26). As depicted from correlation coefficient results, AMH and number of embryos showed positive strong correlation with the number of mature oocytes. This reconfirms the previous result that elevated levels of AMH increased the mature number of oocytes, thus offering more chance to higher number of embryos. This could lead us to the hypothesis that AMH has an indirect effect on the number of embryos (15).

Found strong positive correlation between AMH and the number of mature oocytes in women attending IVF. On the other hand, strong negative correlation was observed between age and E_2_ with mature oocytes. Such negative correlation between age and mature number of oocytes was also reported by (17).

## Conclusion

In conclusion serum AMH levels can be considered the best marker for ovarian reserve and ovarian response during IVF and the best marker reflecting the decline of reproductive aging. Useful clinical applications of AMH seem feasible.
